# Modelling and Experimental Assessment of Inter-Personal Distancing Based on Shared GNSS Observables

**DOI:** 10.3390/s21082588

**Published:** 2021-04-07

**Authors:** Alex Minetto, Andrea Nardin, Fabio Dovis

**Affiliations:** Department of Electronics and Telecommunications (DET), Politecnico di Torino, 10129 Turin, Italy; andrea.nardin@polito.it (A.N.); fabio.dovis@polito.it (F.D.)

**Keywords:** Global Navigation Satellite System, collaborative positioning, collaborative measurements, distance estimation, social distancing, mobile devices

## Abstract

In the last few years, all countries worldwide have fought the spread of SARS-CoV-2 (COVID-19) by exploiting Information and Communication Technologies (ICT) to perform contact tracing. In parallel, the pandemic has highlighted the relevance of mobility and social distancing among citizens. The monitoring of such aspects appeared prominent for reactive decision-making and the effective tracking of the infection chain. In parallel to the proximity sensing among people, indeed, the concept of social distancing has captured the attention to signal processing algorithms enabling short-to-medium range distance estimation to provide behavioral models in the emergency. By exploiting the availability of smart devices, the synergy between mobile network connectivity and Global Navigation Satellite Systems (GNSS), cooperative ranging approaches allow computing inter-personal distance measurements in outdoor environments through the exchange of light-weight navigation data among interconnected users. In this paper, a model for Inter-Agent Ranging (IAR) is provided and experimentally assessed to offer a naive collaborative distancing technique that leverages these features. Although the technique provides distance information, it does not imply the disclosure of the user’s locations being intrinsically prone to protect sensitive user data. A statistical error model is presented and validated through synthetic simulations and real, on-field experiments to support implementation in GNSS-equipped mobile devices. Accuracy and precision of IAR measurements are compared to other consolidated GNSS-based techniques showing comparable performance at lower complexity and computational effort.

## 1. Introduction

The ongoing SARS-CoV-2 (COVID-19) pandemic is repeatedly pushing our communities to reduce social contacts and minimize daily mobility. As a consequence, the paralysis of mobility is dramatically affecting the economy of most of the countries worldwide [1]. In this context, social distancing is of paramount importance to limit the spreading of the infections, and, despite being a challenging task [2], a diffused capability to monitor it could orient strategical decisions. Social distancing refers to the non-pharmaceutical approaches that aim at limiting the frequency and closeness of physical contacts between people [3]. To this end, technical committees at national-scale have established rules to ensure distancing amongst people such as travel restrictions, border control, closure of public places, and warning their citizens to keep a 1.5 m to 2 m distance from each other when they have to go outside [4,5]. When social distancing cannot be guaranteed, *contact tracing* must be ensured to keep track of new possible infections. A huge effort has been invested in the development of application-specific mobile apps, thus targeting mobile devices and their localization data for limiting the spreading of the infection [6,7,8] and supporting an effective digital epidemiological surveillance [9]. In parallel, a set of open ethical issues and challenges have been raised regarding privacy-preservation, scheduling, and incentive mechanisms for the massive implementation of such solutions [10,11]. A comprehensive survey about contact tracing in the COVID-19 pandemic is provided in [6].

In such a scenario, distance estimation technologies can facilitate social distancing and support contact tracing solutions, becoming a key enabler for effective mitigation of the spreading of the infection. Therefore, several technologies have been proposed in the last months not only to assess inter-personal distances but also to cover different aspects of interest such as real-time monitoring for crowd detection and avoiding [3,12,13]. In particular, several radio-frequency wireless technologies [3] and emerging methods exploiting Aritifical Intelligence (AI) and Machine Learning (ML) [12] have been analyzed in recent surveys for distance estimation and social distancing.

Technologies such as Bluetooth [14,15,16], Ultra-Wide Band (UWB) [17], ZigBee [18], and Radio-frequency identification (RFID) [19] have attracted the attention of the research community for their capability of providing ranging measurements between users in dynamic contexts. These ranging technologies are based on Received Signal Strength Indicator (RSSI) or time-based measurements (e.g., time-of-arrival, time-difference-of-arrival, round-trip time) [3]. This means that the feature extracted from the signal, whether it is based on RSSI or time-based, is a reliable measure of the distance only when the signal experiences a direct propagation path (i.e., without relevant obstacles and multipath). As a result, proximity awareness and inter-personal distance estimation can be achieved, but bodies and obstacles can significantly decrease the ranging accuracy. Hence, an accurate distance estimation requires Line of Sight (LOS) visibility among ranging users or a complex propagation model estimation [20]. They also have a limited ranging capability, spanning from short (Bluetooth, RFID) to medium ranges (UWB, ZigBee) [3], a coverage that is not suitable for continuous monitoring of the distance over larger areas.

A different approach for distance estimation relies instead on positioning technologies. An estimate of inter-personal distance can be easily inferred from users’ positions. For instance, users that are able to locate themselves can compute their distance from another user whose coordinates are known. Through this approach, the LOS constraint can be overcome, providing also long-range distance estimation, but privacy concerns may arise due to the explicit position disclosure between users. Technologies such as Wi-Fi [21,22,23], Cellular networks [24], UWB [25], and Global Navigation Satellite System (GNSS) [26] have been used for positioning and can be therefore exploited to this end. Differently from solutions based on Wi-Fi or cellular networks, GNSS is mostly limited to outdoor applications. Nevertheless, 6.7 billion smartphones are estimated by 2023 [27] and GNSS is extremely popular on these devices [28]. With its worldwide coverage, it is certainly the most widespread positioning technology, with more than 6 billion estimated receivers in 2020 [28]. It can be therefore a convenient choice to provide a ubiquitous outdoor distance estimation technology that does not need application-specific hardware. On the other hand, technologies such as UWB, ZigBee, and RFID demand an increased cost to be embedded and additional computational power to perform ranging.

In particular, GNSS has been investigated by researchers to explore cooperative distance estimation [29]. In this framework, the distance between users can be computed indirectly, by leveraging GNSS raw data and a communication channel between cooperating users (namely the *agents*). GNSS-based ranging methods can hence be considered for distance estimation in outdoor environments and in the presence of networked agents. They are based on a ubiquitous technology, which is both (i) readily available in billions of devices and (ii) widespread thanks to a worldwide coverage. Moreover, (iii) it enables short to long-range distance estimation, working also when (iv) LOS between agents cannot be guaranteed (which is even more likely for long-range distances).

Being a promising ranging technology, an assessment of GNSS-based distance estimation was presented in [26] for vehicular applications and, recently, a proof-of-concept demonstrated the feasibility of smartphone-based collaborative positioning based on GNSS inter-agent distances [30].

This study addresses the modeling and experimental assessment of a GNSS-based ranging technique, namely Inter-Agent Ranging (IAR), preliminarily proposed in [31], based on the exchange of a single row of the Direction Cosine Matrix (DCM) (i.e., steering vectors) and the corresponding pseudorange measurement between asynchronous users. Compared to traditional GNSS-based ranging approaches, the IAR technique requires only a single satellite in a common view to building up a collaborative distance measurement among connected devices. In this work, we provide an analytical model for mean and variance estimation of the proposed ranging method. The model is then validated through Monte Carlo simulations and on-field experimental tests. The results presented in this study compare the proposed approach to state-of-the-art GNSS-based ranging methods known in geodesy. All the considered methods share (i–iv) features and they are a straightforward term of comparison. Similarly, long-range urban scenarios are considered as a representative use case to leverage (iii) and (iv). We demonstrate that the proposed method overcomes the performance attainable by a plain absolute position difference while preventing full disclosure of the user position. The IAR technique is found to also be a valid alternative to state-of-the-art GNSS-based ranging approaches that deliver comparable performances while demanding enhanced visibility of common satellites.

### Paper Outline

The rest of the paper is organized as follows: in Section 2, GNSS essentials and the necessary nomenclature and definitions are recalled to support the proposed technique. State-of-the-art GNSS-based ranging methods are summarized and their limitations are described w.r.t. the proposed rationale. GNSS observables are applied to IAR computation in Section 2 as well. In Section 3, the estimation of the IAR is introduced along with the full derivation of a theoretical model to describe its statistical behavior according to the geometry of a dual-agent scenario. In Section 4, a static, controlled environment and real static and kinematic scenarios are investigated by using Commercial Off The Shelves (COTS) GNSS receivers. The theoretical model is validated through synthetically simulated and real experimental data. A comparison of the experimental accuracy w.r.t. the traditional GNSS-based techniques is contextually provided and conclusions are eventually drawn in Section 5.

## 2. Background

In principle, GNSS-based distance estimation can be pursued between embedded ultra-low-cost GNSS receivers which are nowadays integrated into vehicles, mobile devices such as smartphones and tablets, and wearables such as smartwatches. Furthermore, most of the current Location Based Services (LBS)s require data connectivity through which also collaborative approaches can be enabled.

**Figure 1:**
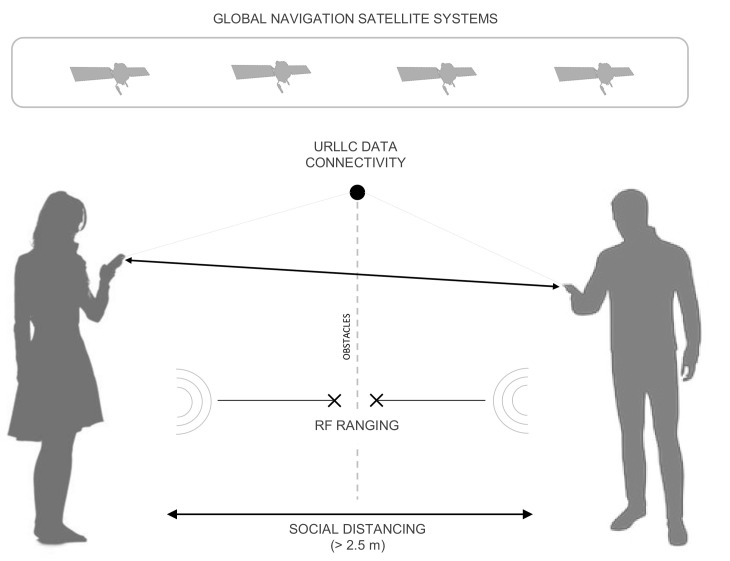
GNSS-based distance estimation among GNSS receivers embedded in networked mobile and wearable devices.

As demonstrated in [30], indeed, the concept of collaborative ranging applied to such a class of devices leverages the exchange of low-level navigation data through current networks (4G/LTE). These applications will be further supported by the next-generations of mobile networks (i.e., 5G-NR Ultra-reliable Low Latency Communication (URLLC) [32,33]). Thanks to the absence of LOS requirements, such a paradigm can also be conceived as a complementary method to more challenging Radio-Frequency (RF) approaches for which obstacles and interference degrade estimation performance, as recalled in Section 1. Figure 1 shows a pictorial representation of distance estimation performed by merging the main enablers of the proposed solution: GNSS and existent mobile network infrastructures.

### 2.1. GNSS Observables and Positioning

The positioning problem in GNSS mainly concerns the determination of a set of coordinates typically referred to as *receiver space state*. The location of a GNSS receiver can be expressed in Cartesian quantities within a given geographic coordinates system (e.g., Earth Centered Earth Fixed (ECEF), Latitude, Longitude, Altitude (LLA)), depending on the target application. The receiver clock offset w.r.t. the in-orbit clocks, namely *user clock bias*, can be contextually estimated, thus providing a common timescale to all the GNSS devices. The overall set of time and three-dimensional coordinates

(1) $$\left[\begin{array}{c} x(t_{k})\\ b(t_{k}) \end{array}\right]={\left[\begin{array}{cccc} x(t_{k}) & y(t_{k}) & z(t_{k}) & b(t_{k}) \end{array}\right]}^{T}$$

defines the *state* of a generic GNSS receiver, namely, the unknowns of the positioning problem at a generic instant $t_{k}$. In (1), $b(t_{k})$ is the clock bias term obtained by multiplying the aforementioned *user clock bias* by the speed of light. To estimate (1), a set of satellite-to-user range estimates are collected at each time instant to solve for a multilateration problem [34]. These satellite-to-receiver ranges are retrieved by the receiver through the estimation of the time-of-flight of the navigation signals received from each visible satellite i∈(1,2,…,N) at a given time instant $t_{k}$. The set of collected measurements is

(2) $$\hat{\rho }\left(t_{k}\right)={\left[\begin{array}{cccc} {\hat{\rho }}^{1}\left(t_{k}\right) & {\hat{\rho }}^{2}\left(t_{k}\right) & \cdots & {\hat{\rho }}^{N}\left(t_{k}\right) \end{array}\right]}^{T}\;.$$

The generic ${\hat{\rho }}^{i}\left(t_{k}\right)$ is referred to as *pseudorange* because of the clock bias component. GNSS pseudorange measurements are altered by a set of impairments affecting the propagation of the navigation signals (e.g., Ionospheric error, Tropospheric error, ephemeris error, relativistic error, etc.). The biases induced by such effects can be modeled and subtracted to the estimated measurements [34]. In this view, a *raw pseudorange measurement* can be defined as

(3) $${\hat{\rho }}^{i}\left(t_{k}\right)=r^{i}\left(t_{k}\right)+b\left(t_{k}\right)+\Sigma_{i}\left(t_{k}\right),$$

where

(4) $$r^{i}\left(t_{k}\right)=||x\left(t_{k}\right)-x_{i}\left(t_{k}\right)\left|\right|$$

is the *satellite-to-user range*. The random variable $\Sigma_{i}\left(t_{k}\right)$ collects all the error contributions affecting the measurement, while the clock bias term, $b(t_{k})$, is common to all the measurements in (2). Once the bias corrections are applied to $\Sigma_{i}\left(t_{k}\right)$, a *corrected pseudorange measurement* can be defined as

(5) $$\rho^{i}\left(t_{k}\right)=r^{i}\left(t_{k}\right)+b\left(t_{k}\right)+\xi_{i}\left(t_{k}\right)\;.$$

The error term, $\xi_{i}\left(t_{k}\right)$, is the User Equivalent Range Error (UERE) which models the independent residual error term not compensated by the aforementioned corrections [35]. It can be assumed to be a zero-mean Gaussian-distributed random variable with a given standard deviation $\sigma^{i}$. Provided a set of corrected pseudorange measurements and satellites’ ephemeris data, an estimate of (1) can be indirectly obtained through an iterative Least Mean Square (LMS) solution

(6) −Δx^(tk)−Δb^(tk)=(HTH)−1HTΔρ(tk).

Indeed, both receiver state and measurement vectors are expressed in incremental notation w.r.t. an approximation point needed for the linearization of the multilateration problem [34]. Consequently, $\Delta \rho (t_{k})$ in (6) is the N×1 column vector obtained as the difference between the measurements and nominal range distances computed between the satellite position and the linearization point. In (6), H is a N×4 Jacobian matrix referred to as *observation matrix* or DCM and defined as

(7) $$H=\left[\begin{array}{cccc} h_{x}^{1} & h_{y}^{1} & h_{z}^{1} & 1\\ h_{x}^{2} & h_{y}^{2} & h_{z}^{2} & 1\\ \vdots & \vdots & \vdots & \vdots \\ h_{x}^{N} & h_{y}^{N} & h_{z}^{N} & 1 \end{array}\right].$$

The axial components in (7) are defined as

(8) $$\begin{array}{c} h_{x}^{i}=\frac{x_{i}\left(t_{k}\right)-x_{0}\left(t_{k}\right)}{r^{i}\left(t_{k}\right)}\\ h_{y}^{i}=\frac{y_{i}\left(t_{k}\right)-y_{0}\left(t_{k}\right)}{r^{i}\left(t_{k}\right)}\\ h_{z}^{i}=\frac{z_{i}\left(t_{k}\right)-z_{0}\left(t_{k}\right)}{r^{i}\left(t_{k}\right)} \end{array}$$

where $x_{0}\left(t_{k}\right),y_{0}\left(t_{k}\right),z_{0}\left(t_{k}\right)$ are the coordinates of the linearization point. The terms in (8) will be referred hereafter to as the *unitary steering vector* $h^{i}\left(t_{k}\right)=\left[\begin{array}{ccc} h_{x}^{i} & h_{y}^{i} & h_{z}^{i} \end{array}\right]$, which is directed towards the *i*-th satellite.

In this work, the bias term $b(t_{k})$ is assumed to be estimated through (6) and compensated for in (5). This allows for modelling the satellite-to-user *estimated range* ${\hat{r}}^{i}\left(t_{k}\right)$ as a zero-mean Gaussian random variable

(9) $${\hat{r}}^{i}\left(t_{k}\right)∼N\left(\;r^{i}\left(t_{k}\right),\;{\left(\sigma^{i}\right)}^{2}\;\right)\;$$

where $\sigma^{i}$ is the standard deviation of the UERE. For the sake of completeness, it is worth remarking that advanced estimation algorithms can be applied to the receiver state estimation, such as Bayesian algorithms (e.g., Extended Kalman Filter (EKF)). Along with these solutions, several integration schemes are typically implemented to fuse auxiliary sensors data (e.g., Inertial Navigation System (INS)) to GNSS in precise positioning applications. However, these aspects do not limit the applicability of the proposed models, and the investigation of these solutions falls out of the scope of this work.

### 2.2. Distance Estimation via GNSS Data

Given the true locations of two independent GNSS receivers, referred to as agent *A* and agent *B*, their true *baseline length* can be expressed as

(10) $$d_{AB}\left(t_{k}\right)≜||d\left(t_{k}\right)\left|\right|=\left|\right|x_{A}\left(t_{k}\right)-x_{B}\left(t_{k}\right)\left|\right|$$

which is, by definition, the Euclidean norm of the *displacement vector* $d(t_{k})$ between the agents [26]. To estimate such a distance using personal GNSS-enabled devices, different approaches can be pursued involving the exchange of different classes of data:

- absolute positioning solutions, as the output of the multilateration algorithm described in Section 2.1;
- raw GNSS measurements and unitary steering vectors obtained through the computation of (7) w.r.t. the current estimated position (instead of the linearization point).

The two approaches lead to the definition of two categories of distance estimation methods, respectively the *Absolute Position Difference* and *Differential GNSS distancing*, which are described in Section 2.2.1 and Section 2.2.2, respectively.

#### 2.2.1. Absolute Position Difference

The practical calculation of (10), named Absolute Position Difference (APD), needs to consider the fact that the positions $x_{A}\left(t_{k}\right)$ and $x_{B}\left(t_{k}\right)$ are results of the estimation process discussed in Section 2.1. Hence, the uncertainty on the estimated positions affects in turn the estimated distance according to

(11) $${\hat{d}}_{AB}\left(t_{k}\right)=\left|\right|{\hat{x}}_{A}\left(t_{k}\right)-{\hat{x}}_{B}\left(t_{k}\right)\left|\right|=d_{AB}\left(t_{k}\right)+\psi_{d}\;,$$

where the generic $\hat{x}\left(t_{k}\right)$ is the estimated solution and $\psi_{d}$ is an error due to the independent positioning errors of the involved receivers. APD is a *naive* approach which can be pursued by exchanging users’ location estimates. In this case, pseudonymization or advanced encryption of the shared data must be implemented to ensure privacy [36].

#### 2.2.2. Differential GNSS Distancing

**Figure 2:**
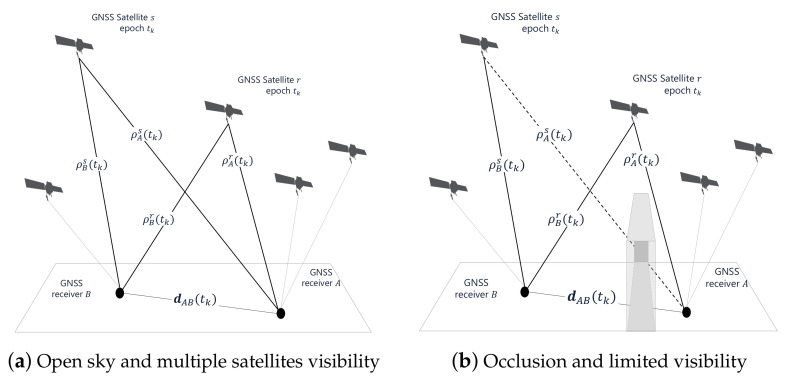
Pictorial representations of scenarios characterized by different visibility conditions of GNSS satellites experienced by independent receivers.

Previous works on the topic investigated a set of algorithms addressing the estimation of (10) within a cooperative framework [26,37,38]. These methods rely on the simultaneous availability of multiple satellites in LOS for both the receivers (as highlighted in Figure 2), hereafter referred to as *shareable satellites*. Differently from APD, they are based on the combination of GNSS observables computed independently by the two agents. In particular, each agent provides the associated unitary steering vectors and pseudorange measurements to ultimately estimate (10).

The displacement vector between the two agents is thus treated as the unknown of the problem, as well as for the individual receiver states in Section 2.1. According to this, raw pseudorange measurements (from shareable satellites) provided by the agent *B* can be aggregated in the measurement vector of agent *A* within the iterative positioning algorithm in charge of performing the inter-personal distance estimation. The observables can be then processed through various differential GNSS methods such as Pseudorange Ranging (PR), Single Differences (SD) or Double Differences (DD) [26]. For code-based measurements in static conditions, it has been demonstrated that the raw-pseudorange based technique (PR) shows the best performance in terms of Root Mean Squared Error (RMSE) w.r.t. more complex methods (i.e., SD and DD ranging) [26].

#### 2.2.3. Applicability

Differently from the aforementioned methods, APD requires the explicit exchange of absolute positioning solutions, thus allowing for any collaborating receiver to be aware of the location of other users. On the other hand, this method does not require shareable satellites between the cooperating agents, being a less demanding technique in terms of sky visibility conditions. PR, SD, and DD methods require instead the exchange of multiple pseudorange measurements between pair of collaborating receivers, as shown in Table 1. Of course, the larger is the amount of disclosed GNSS observables, the higher is the capability of the users to accurately guess the location of collaborating agents. Recent advances in homomorphic encription [36] can be promising solutions to overcome such privacy issues in the aforementioned technique but at the cost of additional complexity. To tackle this aspect, the IAR was proposed in [31] as a ranging method that requires a reduced number of shared GNSS observables. It aims at overcoming the computational complexity issue, reducing the amount of transmitted data, and natively introducing ambiguity in the retrieval of other users’ locations.

**Figure 3:**
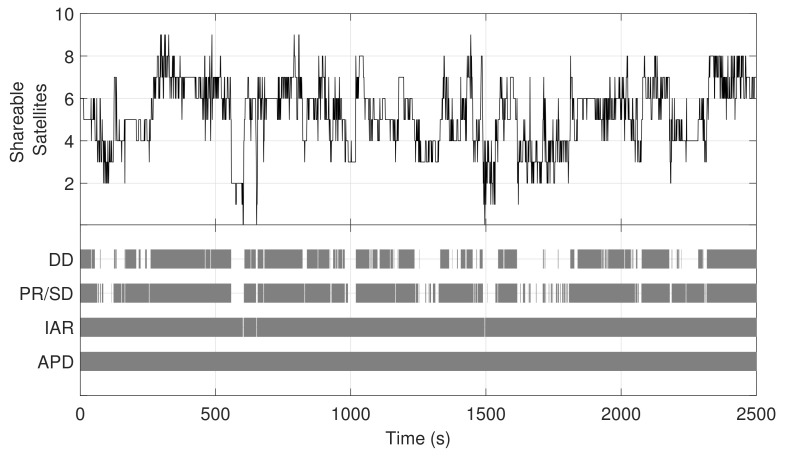
Example of shared satellite visibility for two pedestrian agents through an experimental trajectory in urban environment (upper) and practicability of ranging methods along the time, (bottom) according to Table 1. Post-process data acquired nearby Politecnico di Torino (Turin, Italy), 30 September 2018.

Each receiver employs, in general, a different set of satellites to compute its position. The intersection of the two sets could even contain no elements, making some of the aforementioned techniques unsuitable in harsh environments. Indeed, observing less than three shareable satellites, only APD and IAR can be employed to compute the range between the agents (Table 1). As an example, in the limiting scenario shown in Figure 2b, only one satellite can be shared due to the presence of obstacles that obstruct the LOS. Contextually, applicability analyses performed in urban context have shown a limited availability for traditional differential methods. An example of the number of satellites in common view in a real urban scenario is indeed reported in Figure 3. The data were collected by observing satellite signals through a Global Positioning System (GPS) receiver, which is arguably the most widespread GNSS user equipment.

Throughout the observation window, the limited number of satellites (upper plot) prevents the availability and continuity of the GNSS-based ranging techniques in some time intervals (lower plot). The experimental example shown in Figure 3 provides a hint on the fluctuations in the number of visible GPS satellites experienced by a pair of single-constellation receivers. According to the requirements of Table 1, this motivates the need for complementary techniques for estimating baseline distances among low-cost, personal devices sharing few satellites in LOS.

### 2.3. Inter Agent Ranging

**Figure 4:**
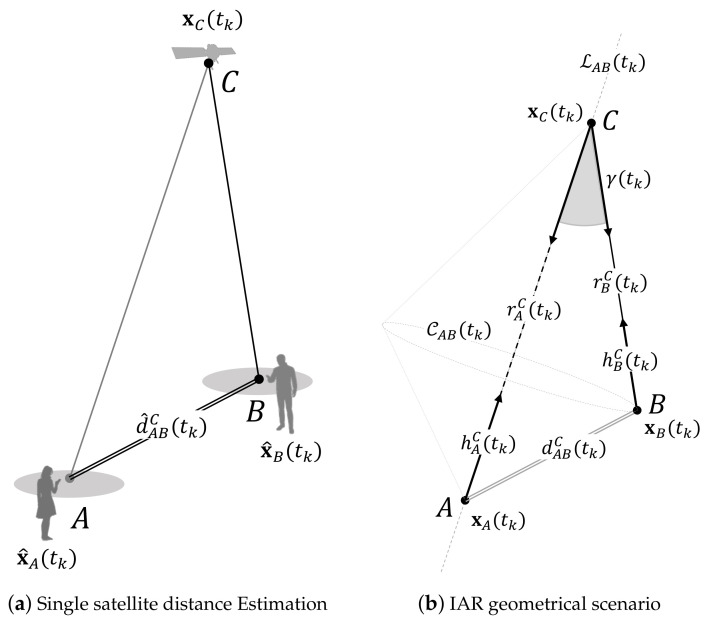
Use case geometry for collaborative distance estimation based on shared GNSS pseudorange measurements.

A simplistic scenario in which two agents, *A* and *B*, observe in LOS a common satellite is recalled in Figure 4. The general use case is shown in Figure 4a, and it can be easily mapped to the formal geometrical arrangement of Figure 4b, which is exploited in the following description.

For an intuitive approach, the position of the shared satellite will be hereafter associated with the letter *C* and identified as the upper vertex of the triangle formed with the agents’ locations. The scheme depicts a static scenario or equivalently the snapshot of a kinematic scenario at a given time instant, $t_{k}$, without any lack of generality. To discuss the theoretical framework of the IAR, the basic geometry is hereafter defined by considering exact measurements and positions, assumed as sides and vertices of this geometrical arrangement. The steering vectors are defined according to (8), pointing towards the shared satellite *C*, whose coordinates are known from the ephemeris broadcasted through the navigation message [42] or retrieved through network connectivity. Given the true satellite-to-user ranges $r_{A}^{C}\left(t_{k}\right)$ and $r_{B}^{C}\left(t_{k}\right)$ and the associated steering vectors $h_{A}^{C}\left(t_{k}\right)$ and $h_{B}^{C}\left(t_{k}\right)$, the IAR can be computed by solving for $d_{AB}^{C}\left(t_{k}\right)$, by means of the Carnot theorem (or law of cosines). The resulting computation is

(12) dABC(tk)=rAC(tk)2+rBC(tk)2−2rAC(tk)rBC(tk)cosγ(tk)rC.

where $\gamma (t_{k})$ is the angle included between the two steering vectors w.r.t. the shared-satellite *C*. It can be computed using the dot product as

(13) $$\gamma \left(t_{k}\right)≜\cos^{-1}\left(h_{A}^{C}\left(t_{k}\right)\times h_{B}^{C}\left(t_{k}\right)\right)=\cos^{-1}\left(h_{A}^{C}\left(t_{k}\right)h_{B}^{C}{\left(t_{k}\right)}^{T}\right)\;.$$

The equivalence in (13) is due to the unitary norm of steering vectors, by definition. Looking at a first basic implementation of this collaborative ranging approach, the agents will be hereafter distinguished as

- *A*, the *aided agent*, which initializes the cooperation asking for the cooperative baseline estimation
- *B*, the *aiding agent*, which supports the aided agent allowing it to gather the cooperative estimate required.

This terminology will be adopted also addressing the aforementioned differential ranging methods.

**Figure 5:**
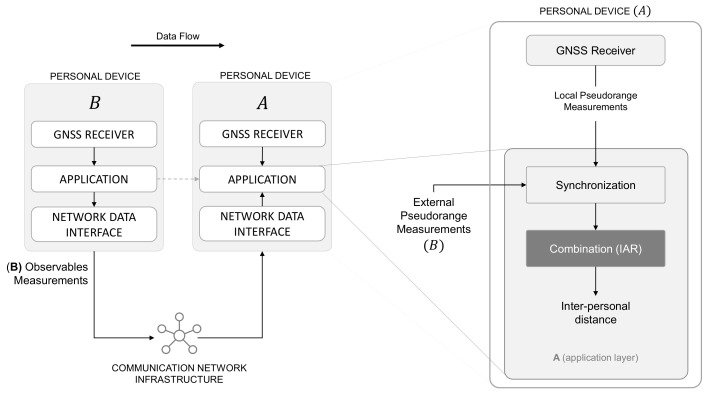
High-level block scheme showing the methodology of the proposed approach.

A more detailed view of the proposed methodology is included in Figure 5, showing how the estimation of the inter-personal distancing (12) is performed within the cooperative framework.

In fact, according to (12), an aided agent *A* that wants to compute the inter-personal distance should (i) measure $r_{A}^{C}\left(t_{k}\right)$ and $h_{A}^{C}\left(t_{k}\right)$, (ii) retrieve from *B* an estimate of $r_{B}^{C}\left(t_{k}\right)$, and (iii) obtain a measure of $\gamma (t_{k})$ from the cooperation with *B*. It is worth noticing that the accomplishment of (iii) can be attained either by sharing $h_{A}^{C}\left(t_{k}\right)$ and let *B* perform the estimation of (13) or by gathering $h_{B}^{C}\left(t_{k}\right)$ from *B* and locally compute (13). A set of variants of the IAR algorithm were proposed in which the roles of the agents can be swapped [31] or re-arranged [43]. However, the formalization of a protocol does not alter the properties of the method and it is therefore out of the scope of this work.

#### 2.3.1. Time Consistency of Input Measurements

**Figure 6:**
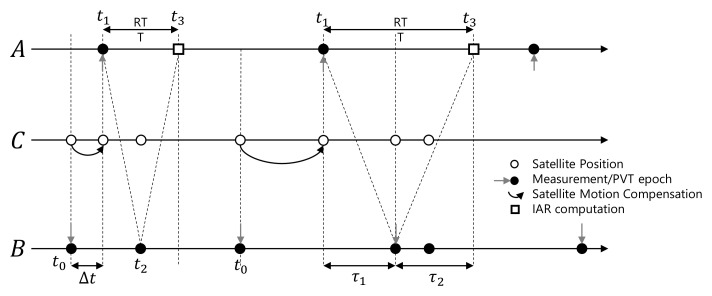
Timing of the exchange of navigation data for the IAR computation between non-synchronous agents. Δt is the delay that must be compensated by the aided agent.

To retrieve an estimate of the baseline through (12), a user needs to obtain a measure of the quantities involved in the IAR computation by establishing the cooperation with an available aiding agent. Figure 6 describes the IAR construction on a temporal axis, assuming that the agents *A* and *B* are not synchronous. The solid dots highlight the measurements epochs at which each receiver estimates the position and updates its measurement vector, both for agent *A* and agent *B*. The variable Δt is the time difference between agents’ measurements epochs, while RTT is the Round Trip Time, which is mainly determined by the communication network. The processing time needed for the computation of (12) can be reasonably neglected.

Let us suppose that agent *A* sends a request, at time $t_{1}$. *A* retrieves the range, $r_{A}^{C}\left(t_{1}\right)$ and computes the steering vector $h_{A}^{C}\left(t_{1}\right)$. *A* is able to send the timestamped steering vector to *B*. Such a request is received by an aiding agent at time $t_{2}=t_{1}+RTT/2$, at a time epoch, which can fall randomly between two measurement epochs of the aiding receiver. The misalignment between agent’s measurement epochs must be taken into account to manage the time-inconsistent measurements of the receivers. In other words, the ranges $r_{A}^{C}\left(t_{k}\right)$ and $r_{B}^{C}\left(t_{k}\right)$ that concur to the computation of (12) must be consistent, even if they are estimated by agents at a different time instant. By knowing the ephemeris and timestamps of the received data, the aiding agent can compensate for the satellite motion (see Figure 6) through

(14) $$\begin{array}{c} r_{B}^{C*}\left(t_{1}\right)=r_{B}^{C}\left(t_{0}\right)h_{B}^{C}\left(t_{0}\right)\;+\;\left[x_{C}\left(t_{1}\right)-x_{C}\left(t_{0}\right)\right] \end{array}$$

(15) $$\begin{array}{c} r_{B}^{C*}\left(t_{1}\right)=\left|\right|r_{B}^{C*}\left(t_{1}\right)\left|\right| \end{array}$$

(16) $$\begin{array}{c} h_{B}^{C*}\left(t_{1}\right)=\frac{r_{B}^{C*}\left(t_{1}\right)}{r_{B}^{C*}\left(t_{1}\right)} \end{array}$$

The closest measurements in time that *B* can use are the measurements taken at time $t_{0}$, which are then compensated for $\Delta t=t_{1}-t_{0}$ seconds by linear regression to make them as consistent as possible with the information provided by *A*. *B* computes the angle $\gamma (t_{1})$ in (13) using the predicted $h_{B}^{C*}\left(t_{1}\right)$ and the received $h_{A}^{C}\left(t_{1}\right)$. At time $t_{3}=t_{1}+RTT$, *A* receives $\gamma (t_{1})$ and $r_{B}^{C*}\left(t_{1}\right)$ and determines $d_{AB}^{C}\left(t_{1}\right)$ through (12). The communication latency only affects the aging of the estimated IAR, which is computed RTT seconds after *A*’s measurement epoch $t_{1}$. This time compensation operation is performed by the “Synchronization” block in Figure 5.

#### 2.3.2. Privacy Issues

It has to be noticed that, even considering the ideal IAR computation (12), the position of the aiding agent at $t_{k}$ can not be retrieved by the aided agent *A* given $\gamma (t_{k})$ and $r_{B}^{C}\left(t_{k}\right)$. The dot product in (13) is not invertible; therefore, agent *A* can obtain only an ambiguous knowledge of the position of *B*. It can assume that its position lies on a circle, referred to as *ambiguity circumference* $C_{AB}$, and shown in Figure 4b. Such *ambiguity circumference* is the locus of the points at distance $d_{AB}^{C}\left(t_{k}\right)$ from the receiver *A* and $r_{B}^{C}\left(t_{k}\right)$ from the satellite *C*. By the aiding side, the only knowledge of the steering vector $h_{A}^{C}\left(t_{k}\right)$ bounds the uncertainty on the location of *A* to a straight line, named *ambiguity line*$L_{AB}$, passing through points $x_{A}\left(t_{k}\right)$, $x_{C}\left(t_{k}\right)$ and the center of $C_{AB}$. This partial protection of the locations of the agents is one of the main advantages of the IAR technique.

## 3. Statistical Modelling for Inter Agent Ranging

Up to now, all the measurements have been considered exact to formalize the basic problem. However, the joint effect of incorrect satellite-to-receiver range measurements and the time-dependent geometry of the observed satellites characterize the distribution of the positioning solution, thus the computation of (12) and (13). Input uncertainties must be taken into account to evaluate the error propagation. Therefore, the inputs of (12) are replaced by the corresponding random variables, obtaining

(17) d^ABC(tk)=RCr^AC(tk)2+r^BC*(tk)2−2r^AC(tk)r^BC*(tk)cosγ^(tk)rC.

where ${\hat{r}}_{B}^{C*}\left(t_{1}\right)$ is computed according to (14).

A key point in the analysis of the IAR measurement as a random variable regards the effects of nonlinearity of (17) on the modeled input random variables (i.e., pseudoranges). Although the computation is performed through a nonlinear equation, previous works assessed that, similarly to Euclidean distance, the error distribution of the IAR can be approximated with a Gaussian distribution when Gaussian inputs are considered and the positioning error is negligible w.r.t. the baseline length [31]. Statistical moments of such a distribution are hereafter derived expanding the range terms according to the pseudorange error model in (9). Satellite-to-receiver ranges are characterized by different standard deviations $\sigma_{A}^{C}$ and $\sigma_{B}^{C}$ for each GNSS receiver and shared satellite *C*. To limit the notation complexity, all the references to the shared satellite *C* and time index $t_{k}$ will be dropped hereafter. Similarly, the range ${\hat{r}}_{B}^{C*}\left(t_{k}\right)$ and the steering vector ${\hat{h}}_{B}^{C*}\left(t_{k}\right)$ will be written as ${\hat{r}}_{B}$ and ${\hat{h}}_{B}$, respectively.

### 3.1. Bias Modelling

**Figure 7:**
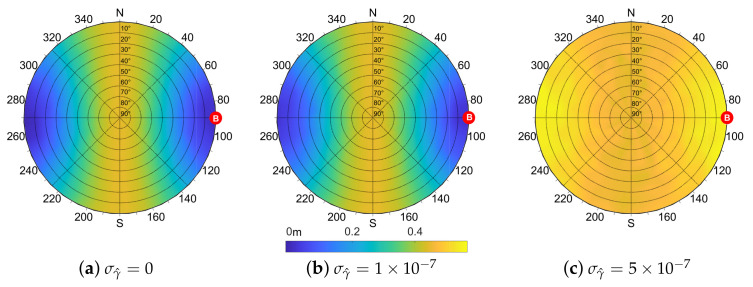
Simulation of the bias of the IAR measurements according to the position of the shared satellite and varying the magnitude of $\sigma_{\hat{\gamma }}$ in $\left[0,1\times 10^{-7},5\times 10^{-7}\right]$ rad, d=100 m, $\sigma_{A}=\sigma_{B}=7.03$ m.

Consider a generic function of *n* random variables

(18) $$Y=g(X_{1},X_{2},...,X_{n})$$

and its Taylor expansion about the mean values $\mu_{X_{1}},\mu_{X_{2}},\ldots ,\mu_{X_{n}}$

(19) $$Y=g\left(\mu_{X_{1}},\mu_{X_{2}},\ldots ,\mu_{X_{n}}\right)+\sum_{i=1}^{n}\left(X_{i}-\mu_{X_{i}}\right)\frac{\partial^{g}}{\partial X_{i}}+\frac{1}{2}\sum_{i=1}^{n}\sum_{j=1}^{n}\left(X_{i}-\mu_{X_{i}}\right)\left(X_{j}-\mu_{X_{j}}\right)\frac{\partial^{2}g}{\partial X_{i}X_{j}}+\ldots$$

where all the partial derivatives of $g(X_{1},X_{2},...,X_{n})$ are evaluated at $\left(X_{1}=\mu_{X_{1}},X_{2}=\mu_{X_{2}},\ldots ,X_{n}=\mu_{X_{n}}\right)$, as well. Truncating the expansion at the first order and applying the expected value to (19), it becomes

(20) $$E\left[Y\right]≃g\left(\mu_{X_{1}},\mu_{X_{2}},\ldots ,\mu_{X_{n}}\right)$$

since the first-order terms are canceled by the expected value operator. The same operations applied to (17) lead to

(21) $$E\left[{\hat{d}}_{AB}\right]≃\sqrt{{\left(r_{A}\right)}^{2}+{\left(r_{B}\right)}^{2}-2r_{A}r_{B}\cos\gamma }$$

which is the definition of the ideal IAR (12), assuming zero-mean distribution of the error affecting the variables ${\hat{r}}_{A}$,${\hat{r}}_{B}$ and $\hat{\gamma }$. According to (21), Equation (17) can be wrongly thought of as an unbiased estimator of $d_{AB}$ since $E\left[{\hat{d}}_{AB}\right]-d_{AB}=0$. However, the statistical behavior of the estimated IAR, obtained through a Monte Carlo simulation campaign, shows generally non-null values of the bias, whose distribution depends on the considered geometry. Figure 7 shows the value of the estimated IAR bias depending on the position of the shared satellite.

Each point of the skyplot corresponds to a pair of azimuth and elevation coordinates of the shared satellite and the associated colormap varies according to the value of the estimation bias. The cooperating agents’ locations are fixed with the aided agent in (0,0) and a distance d=100 m between the two. The standard deviations of the UERE are set to $\sigma_{A}=\sigma_{B}=7.03$ m according to the nominal value of GPS error budget [34]. The plots also show how the value of the bias is dependent on $\sigma_{\hat{\gamma }}$. The non-null bias contributions are attributed to the terms in the Taylor expansion which are truncated due to their higher order. Nonetheless, their value is small if compared to the simulated baseline.

### 3.2. Variance Modelling

The truncation of the Taylor expansion applied to (17) is exploited to obtain a closed-form approximation of the estimated *IAR variance* as well. The variance of a function of multiple random variables is derived as

(22) $$\sigma_{Y}^{2}≜E\left[Y^{2}\right]-E{\left[Y\right]}^{2}≃\sum_{i=1}^{n}\sigma_{X_{i}}^{2}{\left(\frac{\partial g}{\partial X_{i}}\right)}^{2}+\sum_{\begin{array}{c} i,j=1,...,n\\ i\neq j \end{array}}\alpha_{ij}\sigma_{X_{i}}\sigma_{X_{j}}\frac{\partial g}{\partial X_{i}}\frac{\partial g}{\partial X_{i}}$$

where $\alpha_{ij}$ is the *correlation coefficient* [44] of two random variables $X_{i}$, $X_{j}$ defined as

(23) $$\alpha_{ij}=\frac{\mathrm{cov}(X_{i},X_{j})}{\sigma_{A}\sigma_{B}}\;.$$

Consequently, the variance of ${\hat{d}}_{AB}$ can be written as

(24) $$\begin{array}{cc} \sigma_{\hat{d}}^{2}\; & ≃\sigma_{A}^{2}{\left(\frac{{\partial \hat{d}}_{AB}}{{\partial \hat{r}}_{A}}\right)}^{2}+\sigma_{B}^{2}{\left(\frac{{\partial \hat{d}}_{AB}}{{\partial \hat{r}}_{B}}\right)}^{2}+\sigma_{\hat{\gamma }}^{2}{\left(\frac{{\partial \hat{d}}_{AB}}{\partial_{\hat{\gamma }}}\right)}^{2}\\ \; & +2\;\alpha_{AB}\;\sigma_{A}\sigma_{B}\;\frac{{\partial \hat{d}}_{AB}}{{\partial \hat{r}}_{A}}\frac{{\partial \hat{d}}_{AB}}{{\partial \hat{r}}_{B}}+2\;\alpha_{A\hat{\gamma }}\;\sigma_{A}\sigma_{\hat{\gamma }}\;\frac{{\partial \hat{d}}_{AB}}{{\partial \hat{r}}_{A}}\frac{{\partial \hat{d}}_{AB}}{\partial_{\hat{\gamma }}}\\ \; & +2\;\alpha_{B\hat{\gamma }}\;\sigma_{B}\sigma_{\hat{\gamma }}\;\frac{{\partial \hat{d}}_{AB}}{{\partial \hat{r}}_{B}}\frac{{\partial \hat{d}}_{AB}}{\partial_{\hat{\gamma }}} \end{array}$$

which can be easily expressed in a closed-form by computing the partial derivatives. Equation (24) will be referred to as *generalized theoretical formula* for the IAR variance.

Two ranges measured by two different receivers can be considered uncorrelated after the application of the bias corrections in (5). Moreover, the angle γ, computed through the steering vectors, maintains a very poor correlation to a single specific range among those involved in the computation of the positioning solution (6). Thanks to these remarks, it is reasonable to assume uncorrelated variables in (22). In other words, $\alpha_{ij}=0$ when i≠j and (22) becomes

(25) $$\sigma_{Y}^{2}≃\sum_{i=1}^{n}\sigma_{X_{i}}^{2}{\left(\frac{\partial g}{\partial X_{i}}\right)}^{2}\;.$$

The variance of the estimated IAR ${\hat{d}}_{AB}$ can be then approximated as

(26) $$\sigma_{\hat{d}}^{2}≃\frac{1}{d_{AB}^{2}}\left\{\sigma_{A}^{2}{\left[r_{A}-\cos\left(\gamma \right)r_{B}\right]}^{2}+\sigma_{B}^{2}{\left[r_{B}-\cos\left(\gamma \right)r_{A}\right]}^{2}+\sigma_{\hat{\gamma }}^{2}{\left[\sin\left(\gamma \right)r_{A}r_{B}\right]}^{2}\right\}\;.$$

where $d_{AB}$ is as in (12) and highlights the dependency from the true value of the baseline.

In [45], a simplified equation of the IAR variance was computed for two users lying on the same Local Tangent Plane (LTP), assuming null steering error, i.e., an ideal estimation of the angle γ (13). Under the same assumptions, it can be shown that (26) and the solution derived in [45] are equivalent if and only if $r_{A}=r_{B}$ i.e., when the satellite is equidistant from the two peers. The characterization provided in this paper is therefore consistent with the distribution presented in [45], considering that the condition $\sigma_{\hat{\gamma }}=0$ cancels the third term in (26). The model presented in this paper generalizes the empirical derivation provided in [45] to the case where the two users do not lie on the same LTP.

**Figure 8:**
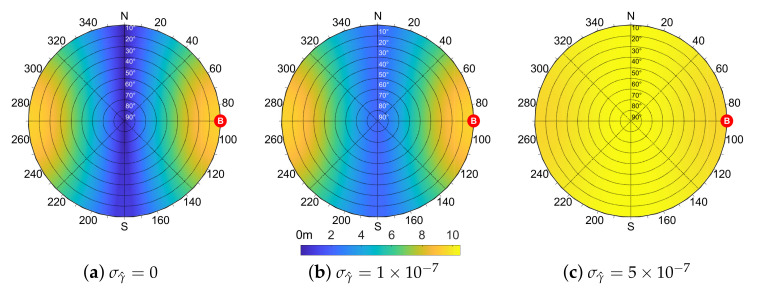
Analytic evaluation of $\sigma_{\hat{d}}$ according to the position of the shared satellite and varying the magnitude of $\sigma_{\hat{\gamma }}$ in $\left[0,1\times 10^{-7},5\times 10^{-7}\right]$ rad, d=100 m, $\sigma_{A}=\sigma_{B}=7.03$ m.

As done for the bias, the values obtained through (26) by varying the location of the shared satellite can be reported on a skyplot. Figure 8 depicts the output of (26) for an aided agent *A* in (0,0) w.r.t. the azimuth and elevation of the shared satellite. The specific position of the aiding agent *B* is highlighted and reasonable values for $\sigma_{A}^{2}$, $\sigma_{B}^{2}$ and $\sigma_{\hat{\gamma }}^{2}$ have been considered.

The skyplots in Figure 8 show the behavior of (26) for different values of $\sigma_{\hat{\gamma }}$. A symmetry perpendicular to the baseline direction is clearly visible for null and small values of $\sigma_{\hat{\gamma }}$. When this term increases due to degraded positioning performances, the IAR standard deviation $\sigma_{\hat{d}}$ also increases assuming a more uniform distribution, as in Figure 8c. From these findings on the IAR error distribution w.r.t. the geometry of the multi-agent system, it can be noticed that the bias has an inverse behavior w.r.t. the standard deviation such that a shared satellite located in a low bias region of the skyplot induces a high variance of the estimated IAR and vice versa.

**Figure 9:**
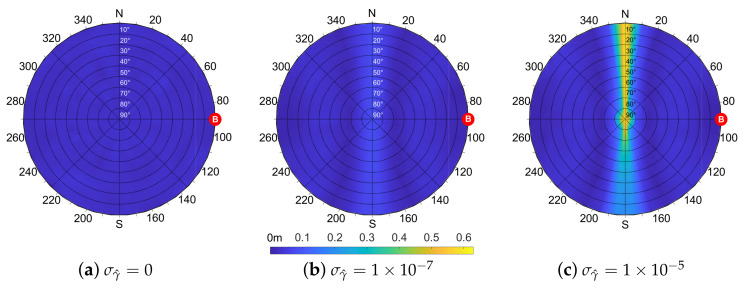
Difference between $\sigma_{\hat{d}}$ estimated from Monte Carlo simulation and from theoretical Formula (26) according to the position of the shared satellite and varying the magnitude of $\sigma_{\hat{\gamma }}$ in $\left[0,1\times 10^{-7},0.5\times 10^{-6}\right]$. d=100 m, $\sigma_{A}=\sigma_{B}=7.03$ m.

The skyplots in Figure 9 are obtained as the difference between simulated and theoretically computed $\sigma_{\hat{d}}$ in the same scenario of Figure 8.

The theoretical formula is proved as a suitable variance estimator, exhibiting a negligible mismodeling error w.r.t. the simulated values in the vast majority of cases (dark-blue areas in Figure 9). Furthermore, the mismatch between the two is always lower than 0.6 m. As the steering error $\sigma_{\hat{\gamma }}$ increases, a non-negligible error can be observed in the areas characterized by high IAR standard deviation values. This is particularly visible in Figure 9c, which is relative to the values of $\sigma_{\hat{d}}$ reported in Figure 8c. This behavior can be reasonably attributed to the neglected cross-correlated terms in (26) that become more relevant as $\sigma_{\hat{\gamma }}$ increases (24), as well as to the truncation of the Taylor expansion.

**Figure 10:**
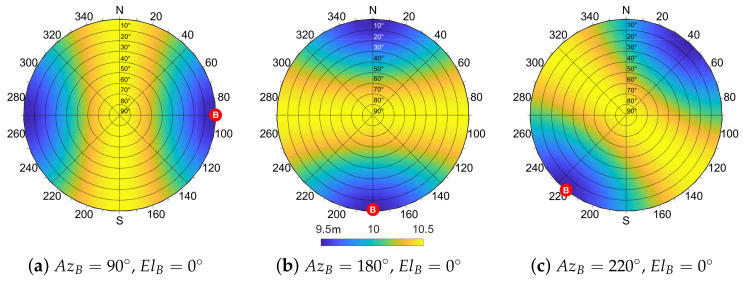
Analytic evaluation of $\sigma_{\hat{d}}$ varying the azimuth angle of the aiding agent *B*, w.r.t. the aided agent *A*. Parameters: $\sigma_{\hat{\gamma }}=0.5\times 10^{-6}$ rad, d=100 m, $\sigma_{A}=\sigma_{B}=7.03$ m.

**Figure 11:**
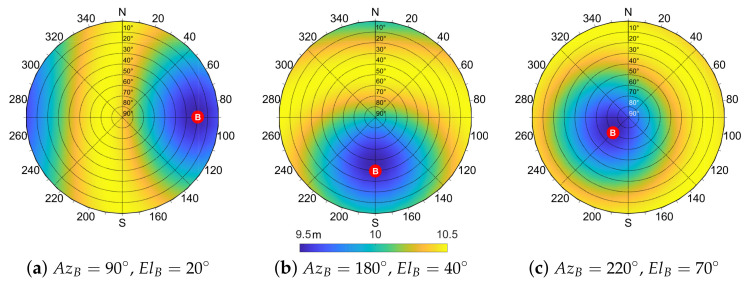
Analytic evaluation of $\sigma_{\hat{d}}$ varying both azimuth and elevation angles of the aiding agent *B*, w.r.t. the aided agent *A*. Parameters: $\sigma_{\hat{\gamma }}=0.5\times 10^{-6}$ rad, d=100 m, $\sigma_{A}=\sigma_{B}=7.03$ m.

The symmetry shown in Figure 8 is preserved in the case of azimuth rotation of the aiding peer, as highlighted by Figure 10. For the sake of completeness, we report also Figure 11, which shows the effect of the movement of agent *B* by varying both elevation and azimuth w.r.t. the agent *A*.

If multiple shareable satellites are available, a suitable model allows the selection of the satellite which is expected to provide the best trade-off between bias and variance of the estimation error. The variance computed through (26) or through the generalized theoretical Formula (24) can therefore influence the choice of the satellite, provided that the aiding agent is able to estimate $\sigma_{B}^{2}$, $r_{B}$, $\sigma_{\hat{\gamma }}^{2}$ and γ, while $\sigma_{A}^{2}$ and $r_{A}$ are estimated by the aided agent.

## 4. Experimental Assessment and Results

In this section, simulation and experimental results will be presented to cross-validate the model for the IAR error previously introduced and to demonstrate the effectiveness of the IAR technique w.r.t. other GNSS-based methods for distance estimation.

### 4.1. Simulated Performance of IAR vs. APD

As discusses in Section 2, the IAR method is able to provide a baseline estimation when the number of simultaneously visible satellites does not guarantee applicability of other GNSS-based ranging techniques (i.e., PR, SD, DD), with the exception of APD (Figure 3). According to these remarks, the IAR method has been compared to APD, exploiting the theoretical model introduced in Section 2 for the computation of its standard deviation. A similar theoretical model for the error distribution of APD is out of the scope of this work. Thus, the variance of the estimated IAR, computed through the theoretical model, is compared to the statistical moments of APD obtained by means of a Monte Carlo simulation, varying the baseline length and the position of the shared satellite (determined by the azimuth ϕ and the elevation θ).

For the sake of completeness, the standard deviation of IAR $\sigma_{\hat{d}}\left(\theta ,\phi \right)$ and APD $\sigma_{APD}\left(\theta ,\phi \right)$ are compared, as well as their mean values ($\mu_{\hat{d}}\left(\theta ,\phi \right)$ and $\mu_{APD}\left(\theta ,\phi \right)$ respectively), through the computation of a gain factor. Gain metrics are computed for all the possible coordinates of a shared satellite by averaging on its azimuth and elevation values, as

(27) $$G_{\sigma }=\frac{1}{N}\sum_{\theta ,\phi }10\log_{10}\left(\frac{\sigma_{APD}\left(\theta ,\phi \right)}{\sigma_{\hat{d}}\left(\theta ,\phi \right)}\right)$$

(28) $$G_{\mu }=\frac{1}{N}\sum_{\theta ,\phi }10\log_{10}\left(\frac{|\mu_{APD}\left(\theta ,\phi \right)-d|}{|\mu_{\hat{d}}\left(\theta ,\phi \right)-d|}\right)$$

**Figure 12:**
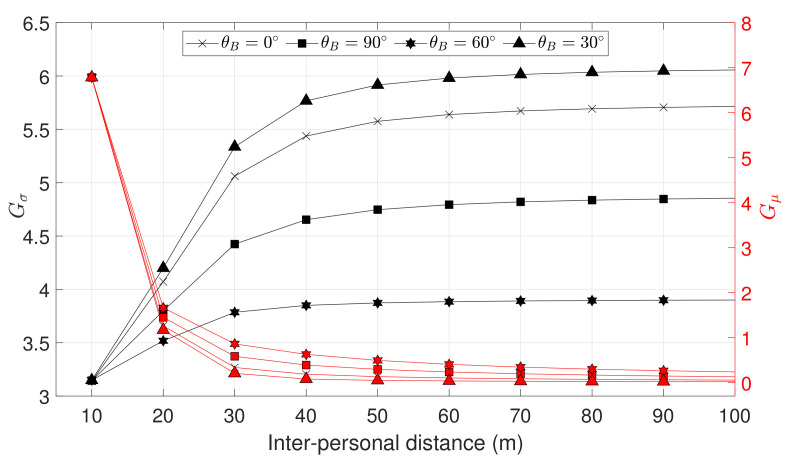
Average bias and variance gain using the IAR method w.r.t. APD. Results shown for different values of elevation of the agent *B*, varying the baseline length.

Figure 12 reports the values of (27) and (28) w.r.t. the baseline length, for different elevation angles of the receiver *B*. The standard deviation of the IAR estimate $\sigma_{\hat{d}}$ always shows a positive gain value for every baseline length, saturating for baseline lengths above 100 m. The bias gain $G_{\mu }$ is positive as well and decreases asymptotically to 0 for long baselines, where the performances of APD and IAR are equivalent.

This analysis confirms the preliminary results presented in [45], emphasizing the use of the IAR as a valuable alternative to the APD and also showing clear advantages in terms of variance of the output measurements.

### 4.2. Experimental Framework in Controlled Static Environment

**Figure 13:**
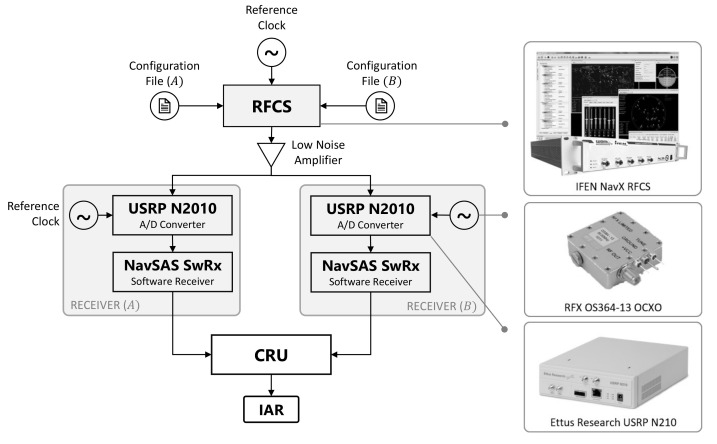
Simulation test-bench for IAR performance assessment in a controlled environment.

A further assessment of the statistical model introduced in Section 3 can be done in a controlled environment, exploiting real signals, but preventing the non-modeled impairments from affecting the dataset as in an on-field scenario (i.e., multipath and atmospheric impairments). Two static receivers have been simulated by means of IFEN^™^ NavX professional Radio Frequency Constellation Simulator (RFCS) (IFEN GmbH, Poing, Germany), as shown in the scheme in Figure 13. The two GNSS receivers tackled and performed the positioning autonomously and asynchronously, based on independent runs of the same reference oscillator RFX OS364-13 Oven Controlled Xtal Oscillator (OCXO) (RFX Ltd., Livingston, UK). For the presented results, agents have been simulated with fixed baseline distance d=126.5962 m, as obtained from the Euclidean distance of LLA coordinates reported in Table 2.

The same scenario was generated at different times of the day to investigate different relative geometries of the visible satellites. Baseband raw samples of incoming navigation signals were recorded through a general-purpose front-end [46], i.e., Ettus Research^™^ Universal Software Radio Peripheral (USRP) N210 (National Instruments Corporation, Austin, TX, US). Relevant configuration parameters are included in Table 3. The output binary file (.bin) including the stream of samples was then post-processed by a proprietary MATLAB^®^ GNSS fully-software receiver, i.e., NavSAS Software Receiver (NavSAS SwRx) (Politecnico di Torino, Turin, Italy), in order to extract GNSS observables and positioning solutions. The software receiver was intended as a flexible GNSS receiver configured according to the parameters reported in Table 4.

According to Section 2.3.1, asynchronous GNSS receivers provided observables and positioning data at different epochs depending on their acquisition, tracking, and Position Velocity and Time (PVT) computation algorithms. The time misalignment, Δt, between the measurements epochs was hence compensated, according to (14). Detrended pseudorange measurements have been assumed as ergodic random processes; therefore, their variance was reliably estimated by means of a second-order discrete derivative [47]. Time variance was hence taken into account in the form of sample variance estimated from such derived time series and used for the evaluation of the proposed theoretical formula. A dedicated MATLAB^®^ script was implemented in the aforementioned fully-software receiver to process the observables obtained at the previous step. Such a script referred to as Collaborative Ranging Unit (CRU), acted as a dual-receiver IAR simulator.

#### Validation of the Theoretical Model within a Controlled Environment

**Figure 14:**
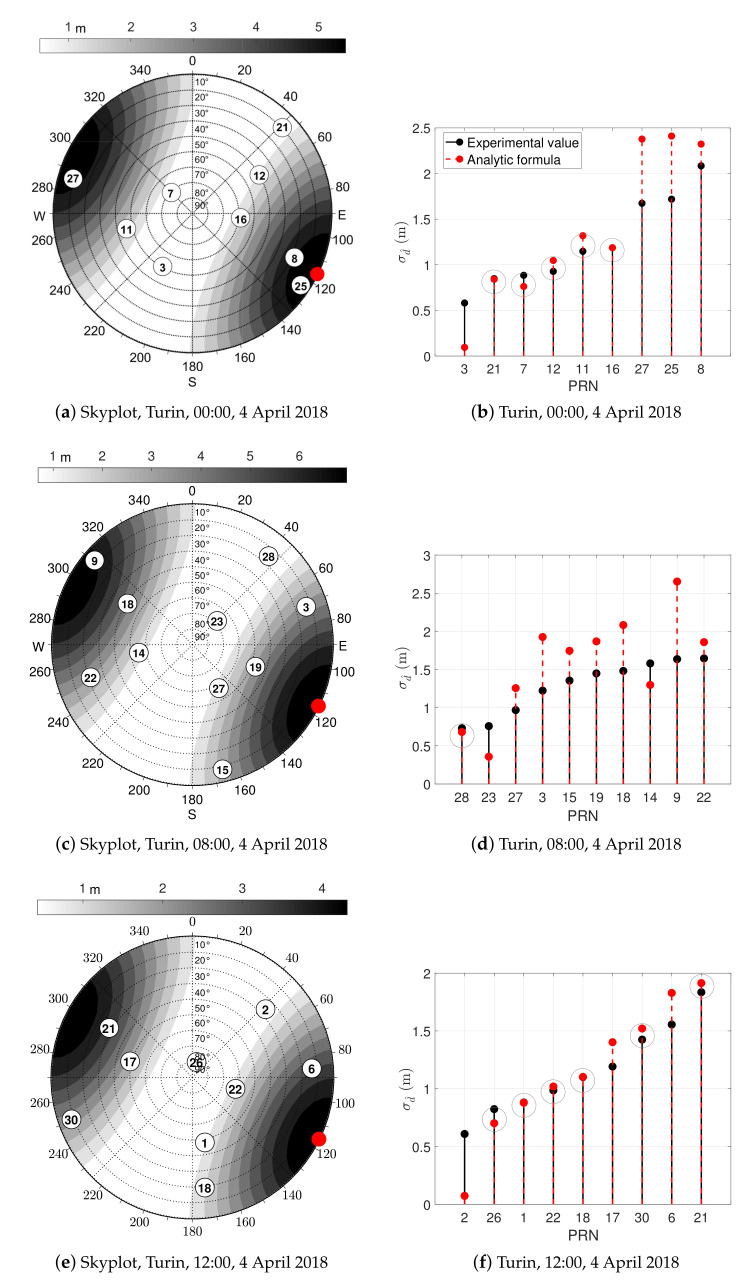
Skyplots (**a**,**c**,**e**) showing the analytic $\sigma_{\hat{d}}$ (color-scale) along with the superposition of satellites azimuth and elevation generated by the RFCS. Ranking plot (**b**,**d**,**f**) of all the shareable satellites based on the experimental standard deviation and the interpolation of the analytic model.

The theoretical Formula (26) for the computation of the IAR standard deviation is adopted with experimentally-estimated values for the assessment and characterization of the standard deviation of the IAR measurements. Qualitative and quantitative analyses are provided about $\sigma_{\hat{d}}$ in Figure 14, computed for the available satellites for three independent experiments. A set of best matches is highlighted in each experiment by gray circles when the difference between experimental and estimated values is below the 5% of the measured quantity. As an example, it is worth noticing that Pseudo Random Noise code (PRN) 21 and PRN 16 returns a valuable match with the analytic extrapolation in Figure 14b. For other PRNs, the difference between measured and theoretically computed $\sigma_{\hat{d}}$ is comparable to the values reported in the skyplots of Figure 9.

### 4.3. Experimental Framework with COTS GNSS Receivers

The results from on-field tests are hereafter exploited to validate the theoretical findings of Section 3, as well as to provide a valuable comparison among state-of-the-art, GNSS-based ranging methods and the proposed solution. By exploiting raw pseudorange measurements provided by COTS receivers, two experimental campaigns have been pursued investigating the IAR computation in a real environment.

- *Static urban scenarios*, where both the aided and aiding receivers performed static positioning at different baseline lengths. The set of selected locations is representative of a mixed urban environment including building occlusions, limited sky visibility, and possible multipath phenomena. However, the impact of environmental parameters cannot be effectively controlled and only the distance from the lab location is considered to discriminate the tests.
- *Semi-dynamic urban scenario*, in which the aiding receiver was kept static while the aided receiver was moved by walking in a dense urban scenario connecting the locations of the static test campaign.

**Figure 15:**
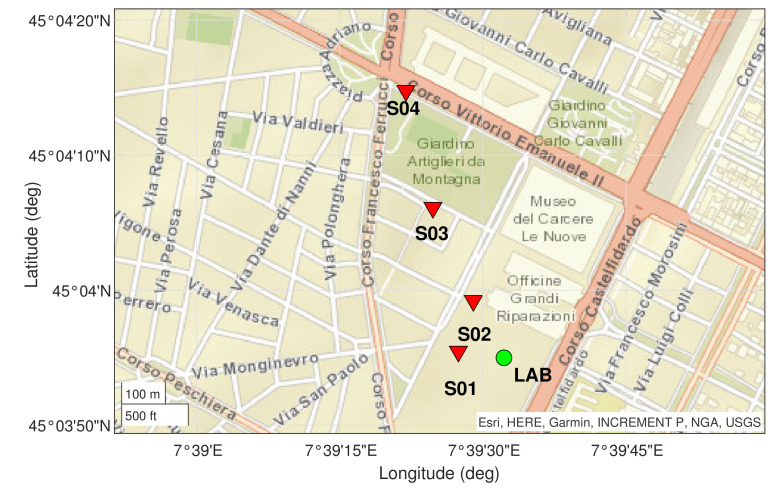
Scenarios map of static reference locations. Dynamic trajectories were achieved by connecting the locations from S01 to S04.

The choice of specific GNSS mass-market receivers does not limit the generality of the results. The tests locations were set in Turin (see Figure 15) and the details are summarized in Table 5.

**Figure 16:**
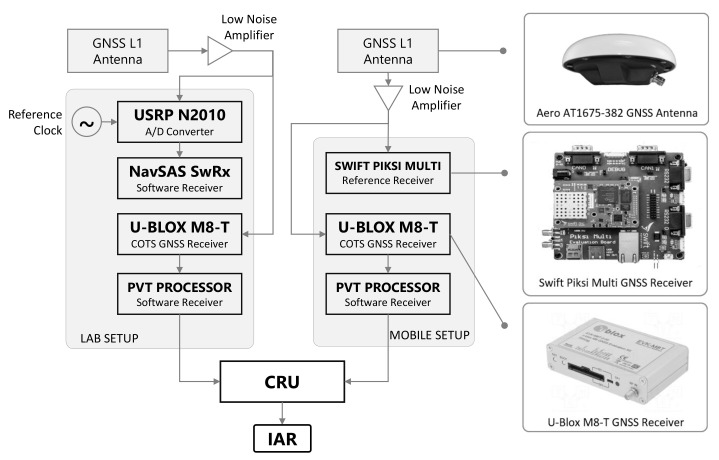
Simulation test-bench for performance assessment of GNSS-based ranging methods in real environment.

The setup of the experiment, shown in Figure 16, included two COTS u-Blox NEO-M8T receivers (u-blox AG, Thalwil, Switzerland) identically configured. The navigation solution rate was set to 1 Hz provided through multi-constellation positioning. The first receiver was connected to a georeferenced geodetic antenna located at the rooftop of LINKS Foundation (Turin, Italy) $45^{\circ }3^{\prime }54.9972^{\prime \prime }$ N, $7^{\circ }39^{\prime }32.2128^{\prime \prime }$ E, 311.804 m. The second receiver was instead moved along with a dual-frequency Swift Piksi Multi receiver used as reference. They were both connected to the same Aero Antenna AT1675-382 (AeroAntenna Technology Inc., Chatsworth, CA, USA).

No synchronization was established between the two u-Blox receivers: pseudorange measurements and related positioning solutions were collected according to the independent onboard clocks as performed for the controlled experiment. The logged raw pseudorange measurements obtained from the M8Ts were re-processed offline to determine a plain LMS positioning solution considering only GPS satellites. The inter-personal distances were then computed according to methods mentioned in Section 2.2 and compared with our proposed IAR technique. The results presented in this section were obtained by selecting output inter-receiver ranges characterized by a reasonable time difference Δt between the measurements of the two collaborating receivers. By considering high values of Δt, indeed the correction provided through (14) does not effectively compensate for the time-inconsistency of the data. Therefore, the maximum Δt considered for the experiment was 50 ms, which guarantees a considerable margin for the misalignment of different high-rate positioning solutions provided by collaborating COTS receivers [48].

#### 4.3.1. Validation of the Theoretical Model Using COTS Receiver

**Figure 17:**
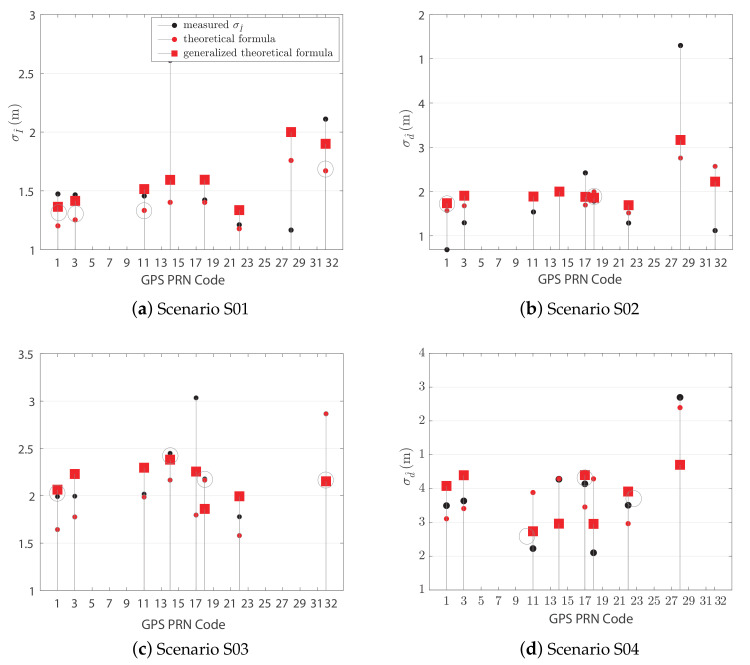

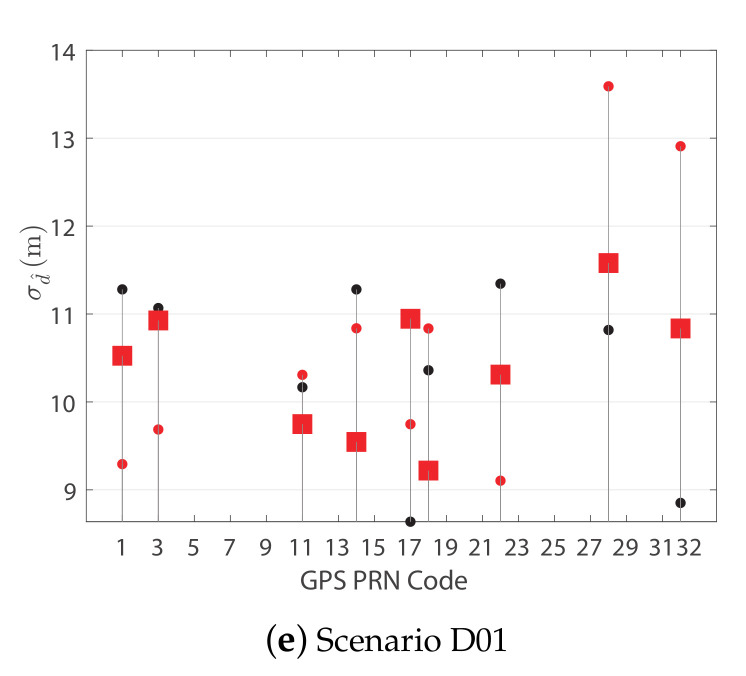
IAR variance estimated from experimental data compared to theoretical models. Circled values regards relevant matches between model and real measurements.

The statistical models presented in Section 3 are further investigated through a comparison w.r.t. experimental data. They are used to obtain an estimate of the IAR variance considering the satellite constellation and the receiver positions during the experiments. Given the position of the users *A*, *B* and of the satellite *C* at a certain time instant, it is possible to estimate the resulting IAR variance by knowing the variances of the random variables involved in the IAR estimation (17) (i.e., $\sigma_{A}^{2}$, $\sigma_{B}^{2}$, $\sigma_{\hat{\gamma }}^{2}$). The baseline is evaluated through a IAR computation based on observations from the experiments, and its variance is compared to the variance predicted by (26) in the same conditions. The satellites’ movements along the tests duration induce a change in the IAR variance (26) as well. However the difference between the minimum and maximum variance computed from (26) within the test duration is always below $10^{-5}$ m in each experiment, and it can be reasonably neglected. As a consequence, the variance of the experimental IARs is compared to the mean of the set of values computed using (26) over the observation window. The result is shown in Figure 17, where the theoretical variance is proved to match the behavior of the measured variance for the majority of the PRNs. The gaps between the two variances are summarized in Table 6, averaging among PRNs.

Equation (26) models the variance of the IAR computed from the experimental datasets with a good approximation. However, the observations of the random variables (${\hat{r}}_{A}$, ${\hat{r}}_{B}$, $\hat{\gamma }$) are collected throughout time and the satellite movement throughout the experiment’s duration introduces correlation among the random variables. Such a correlation can be modeled by the coefficients introduced in (22), which can be estimated, on *M* observations, as

(29) $${\hat{\alpha }}_{ij}=\frac{1}{M-1}\sum_{m=1}^{M}\left(\frac{X_{i,m}-\mu_{X_{i}}}{\sigma_{X_{i}}}\right)\left(\frac{X_{j,m}-\mu_{X_{j}}}{\sigma_{X_{j}}}\right)$$

where $X_{i,m}$ is the *m*-th observation of the random variable $X_{i}$. The correlation coefficients estimated through (29) are employed in the generalized theoretical Formula (24) to obtain a refined estimation of the IAR variance. As shown in Figure 17, taking into account the correlation between the measurements improved the match between the theoretical and experimental values. Table 6 summarizes a mismatch reduction between 6.8% and 35%, depending on the experimental conditions.

#### 4.3.2. Accuracy and Precision of the Ranging Methods

**Figure 18:**
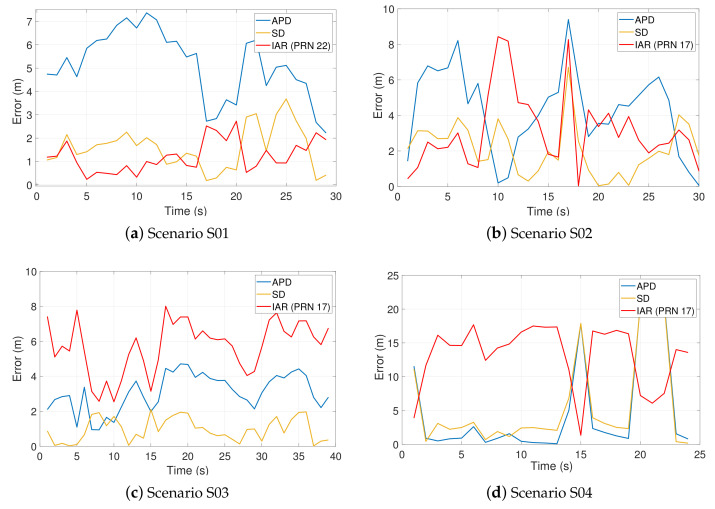
Inter-personal distance estimation error from static experiments. Comparison among relevant state-of-the-art techniques in Table 1. The presented timespans correspond to the longest periods of each experiment during which Δt<50 ms was verified.

Figure 18 presents the accuracy performance observed in the scenarios listed in Table 5, in which the IAR measurements errors are compared with the techniques presented in Section 2.2, along a subset of epochs. DD is not reported due to a lack of shareable satellites, while the PR method proposed in [26] is dropped due to its strong similarity to SD. It is worth noticing that a proper satellite choice returns remarkable ranging performance overcoming on average both APD and SD methods in scenarios S01 and S02 of Table 5. The satellite that minimizes the estimated IAR variance is chosen a priori through (26) and the resulting baseline estimation is compared to other methods. Standard deviations estimated along the epochs for each experiment are reported in Table 7. It can be shown that the IAR presents comparable performance w.r.t. SD, and it outperforms APD in S01, S04, and D01.

**Figure 19:**
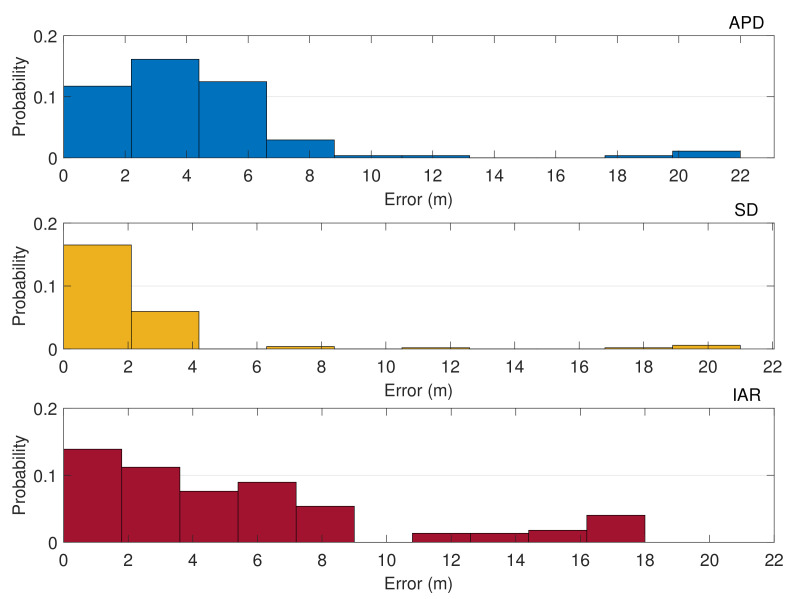
Histograms of the estimation errors for inter-personal distances obtained through APD, SD, and IAR in a static urban scenario.

The histograms in Figure 19 collect the error statistics of each estimation technique for all the experimental static scenarios. This allows for a general evaluation of the distance estimation accuracy in a mixed-urban scenario by in parallel assessing the remarkable performance of the proposed IAR. Most of the estimation errors are indeed lower than 8 m for the APD method, while better results are observed for SD method whose errors are mostly lower than 4 m. A different trend is instead observed for IAR which shows a considerable number of error occurrences in the range 0–8 m. IAR accuracy is mostly penalized by a poor cancellation of common errors w.r.t. differential methods (i.e., SD), and this is evident as the baseline distance increases. However, despite a general lower accuracy, it is worth recalling that IAR does not require an explicit disclosure of the positioning data as for APD or the possibility to indirectly estimate it as for SD. Furthermore, the proposed IAR method relies on a less demanding sky visibility requirement (see Table 1) w.r.t. SD and w.r.t. differential methods in general.

**Figure 20:**
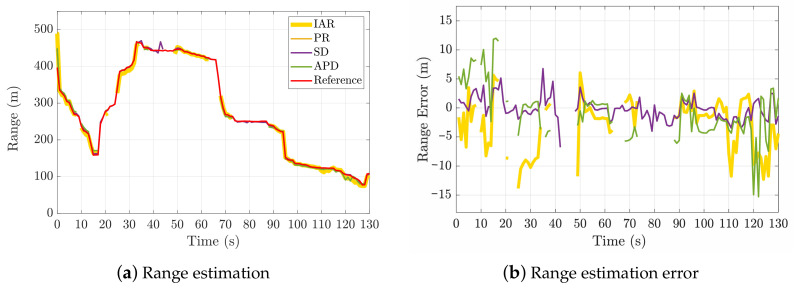
Results of inter-personal distance estimation in dynamic scenario (D01). Comparison of the proposed IAR technique and state-of-the-art GNSS-based ranging methods. Maximum Δt considered 50 ms.

The dynamic scenario D01 described in Figure 20 shows that the IAR measurements (which require a single shared satellite) have an appreciable match to the true baseline w.r.t. other techniques requiring more common satellite in view.

The baseline error of the estimated IAR shown in the lower plot appears to be very sensitive to the dynamics of the multi-agent system. This experimental result is in accordance with the spatial distributions of IAR bias and variance presented in Section 2.

## 5. Conclusions

The technological effort in SARS-Cov-2 pandemic has been mostly focused on contact tracing through short-range proximity sensing, but the emergency has in parallel highlighted the relevance of social distancing to counteract the infection spreading. Besides supporting the capability of monitoring, assessing the inter-personal distance could provide vital information to decision-makers about citizens’ behavior. However, a measure of this quantity is not easily affordable without disclosing positioning data of GNSS-enabled personal devices or enhancing the latter with dedicated ranging hardware. Therefore, a low-complexity distance estimation algorithm, namely IAR, is modeled and assessed in this work for the estimation of the inter-personal distance in non-LOS conditions among mobile personal devices equipped with low-cost and ultra-low-cost GNSS receivers. The method leverages the exchange of a limited set of raw GNSS data between networked receivers and the simultaneous visibility of a common GNSS satellite. A statistical model for the inter-personal distance estimation through IAR has been provided and experimentally validated. The performance of the algorithm has been compared with state-of-the-art GNSS-based ranging methods in terms of measurement variance and bias, through simulated and on-field experimental campaigns. The IAR cooperative solution shows on average better performance w.r.t. the APD and comparable performance w.r.t. iterative methods such as SD and PR, which require, however, multiple shared satellites and allow for indirectly disclosing the position of the aiding agents. IAR can hence be considered as a complementary or alternative technique to similar solutions whenever a limited number of satellites are available for computation. Moreover, similarly to the aforementioned methods, IAR provides a measure of inter-personal distance utilizing only GNSS data and avoiding further implementation of additional ranging sensors.

## Figures and Tables

**Table 1:** GNSS-based ranging algorithms.

Acronym	Ranging Method	Min. No. of Shareable Satellites	References
APD	Absolute Position Difference	0	[26]
IAR	Inter-agent Range	1	[31]
PR	Raw Pseudorange	3	[26]
SD	Single Difference	3	[26,31]
DD	Double Difference	4	[26,30,38,39,40,41]

**Table 2:** LLA coordinates of simulated agents in a controlled environment.

Agent	Latitude (deg)	Longitude (deg)	Altitude (m)
Aiding	45.065274	7.658969	311.973
Aided	45.064775	7.650414	311.635

**Table 3:** Ettus Research USRP N210.

Parameter	Value/Unit
Carrier frequency	1575.42 MHz
Intermediate frequency	0 Hz
Sampling Frequency	5 MHz
Quantization bits	16
Sampling mode	IQ
Reference clock	External
Gain	38 dB

**Table 4:** NavSAS Software Receiver.

Parameter	Value/Unit
Constellation/Signal	GPS/L1
No. of Channels	10
Integration time	20 ms
Doppler step	125 Hz
Coherent accumulations	5
Freq. Lock Loop (FLL) time	10 ms
Position output rate	1 Hz

**Table 5:** Test scenarios for GNSS-based ranging (LLA coordinates of the aided peer).

Name	Latitude (deg)	Longitude (deg)	Baseline (m)	Test Duration (s)
S01	45.065407	7.657622	100	565.1
S02	45.066450	7.658056	126	640.2
S03	45.068365	7.656880	360	606.2
S04	45.070769	7.656095	630	622.6
D01	Dynamic	Dynamic	100–650	$1.3866\times 10^{3}$

**Table 6:** Comparison between theoretical IAR variance and measured variance. The generalized theoretical Formula (24) and the theoretical formula with the assumption of null cross-correlation (26) are compared.

Experiment Identifier	Gap of Theoretical Formula	Gap of Generalized Theoretical Formula	Gap Reduction
S01	0.362 m	0.322 m	11.1%
S02	0.749 m	0.698 m	6.8%
S03	0.151 m	0.099 m	34.4%
S04	1.049 m	0.698 m	33.5%
D01	1.683 m	1.094 m	35.0%

**Table 7:** Estimated standard deviations of ranging techniques within real experiments in urban environments.

Test Scenario	IAR (Single Satellite)	APD	SD
S01	0.5490 m	1.4469 m	0.9080 m
S02	2.4344 m	2.3830 m	1.4381 m
S03	1.4763 m	1.0544 m	0.6702 m
S04	4.6664 m	7.3327 m	6.8653 m
D01	2.8590 m	3.5012 m	2.8954 m

## Data Availability

Not applicable.
